# A Rare Case of Cystic Lymphangioma of the Scrotum: A Case Report

**DOI:** 10.1002/ccr3.71127

**Published:** 2025-10-20

**Authors:** Sen Yan, Yi‐Feng Jiang, Dan Chen

**Affiliations:** ^1^ Department of Urology Chengdu Seventh People's Hospital, Affiliated Cancer Hospital of Chengdu Medical College Chengdu China

**Keywords:** cystic lymphangioma of the scrotum, diagnosis, MRI examination, surgical treatment, ultrasound examination

## Abstract

Urologists need to have the ability to distinguish rare benign tumors in the scrotum. This article aims to highlight the rarity of scrotal lymphangiomas and the necessity of surgical treatment, and the key to preventing recurrence lies in the complete removal of the lesion.

## 1 Introduction

Lymphangiomas are characterized as abnormal formations of the lymphatic system [1]. Lymphangiomas are categorized as either congenital or acquired. Lymphangiomas can occur anywhere in the body. The majority (95%) are found in the neck and axilla, and the remaining 5% occur in locations such as the mediastinum, bone, retroperitoneum, kidney, and liver [2]. There are few cases of scrotal lymphangioma that have been reported [3, 4, 5]. Due to its rarity, diagnosis and treatment can be challenging. Herein, we reported a rare case of cystic lymphangioma in the scrotum to contribute to the knowledge of proper management of this condition.

## 2 Presentation of Case

### 2.1 Case History

A 13‐year‐old male presented with perineal pain that began 3 weeks prior to presentation and had been aggravated for 3 days. The patient experienced paroxysmal dull pain and discomfort at the root of the right scrotum without obvious cause 3 weeks prior to presentation. The pain intensified during exercise and local compression. As time went by, the patient's pain was further aggravated and presented as persistent distending pain. A mass could be palpated locally with obvious tenderness. These discomforts seriously affect the patient's daily activities and lives. However, the patient had no past illnesses and no specific personal or family history.

### 2.2 Physical, Laboratory and Imaging Examination

**FIGURE 1:**
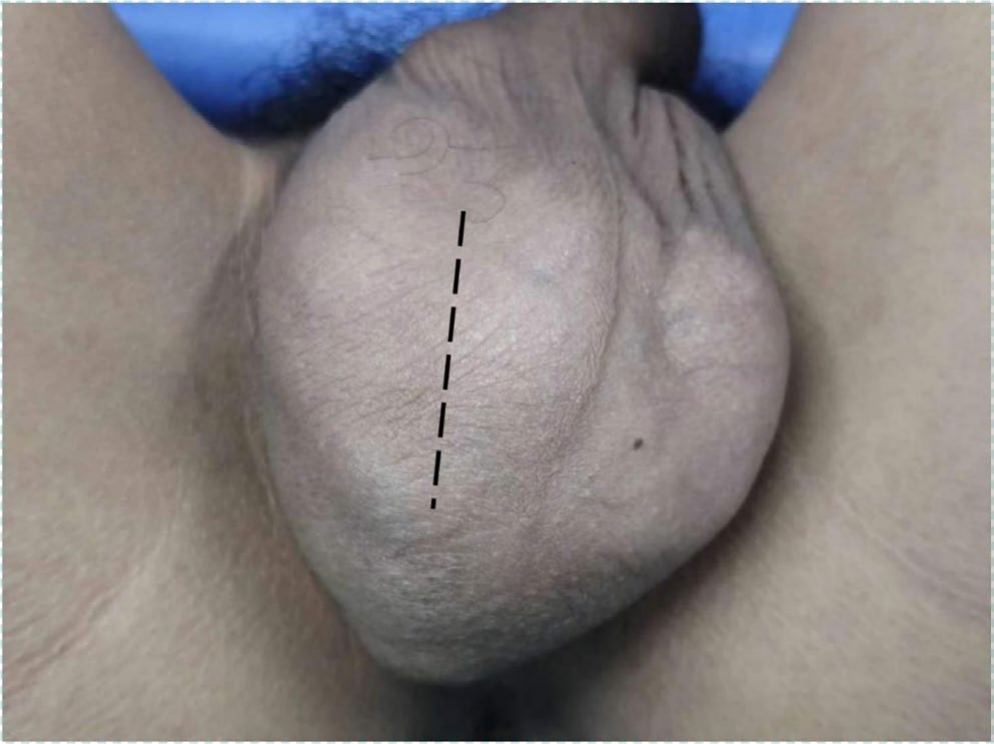
Physical examination. An irregular bulge on the right scrotum is pressing on the left scrotum. The area marked by the dotted line represents the location of the surgical incision.

**FIGURE 2:**
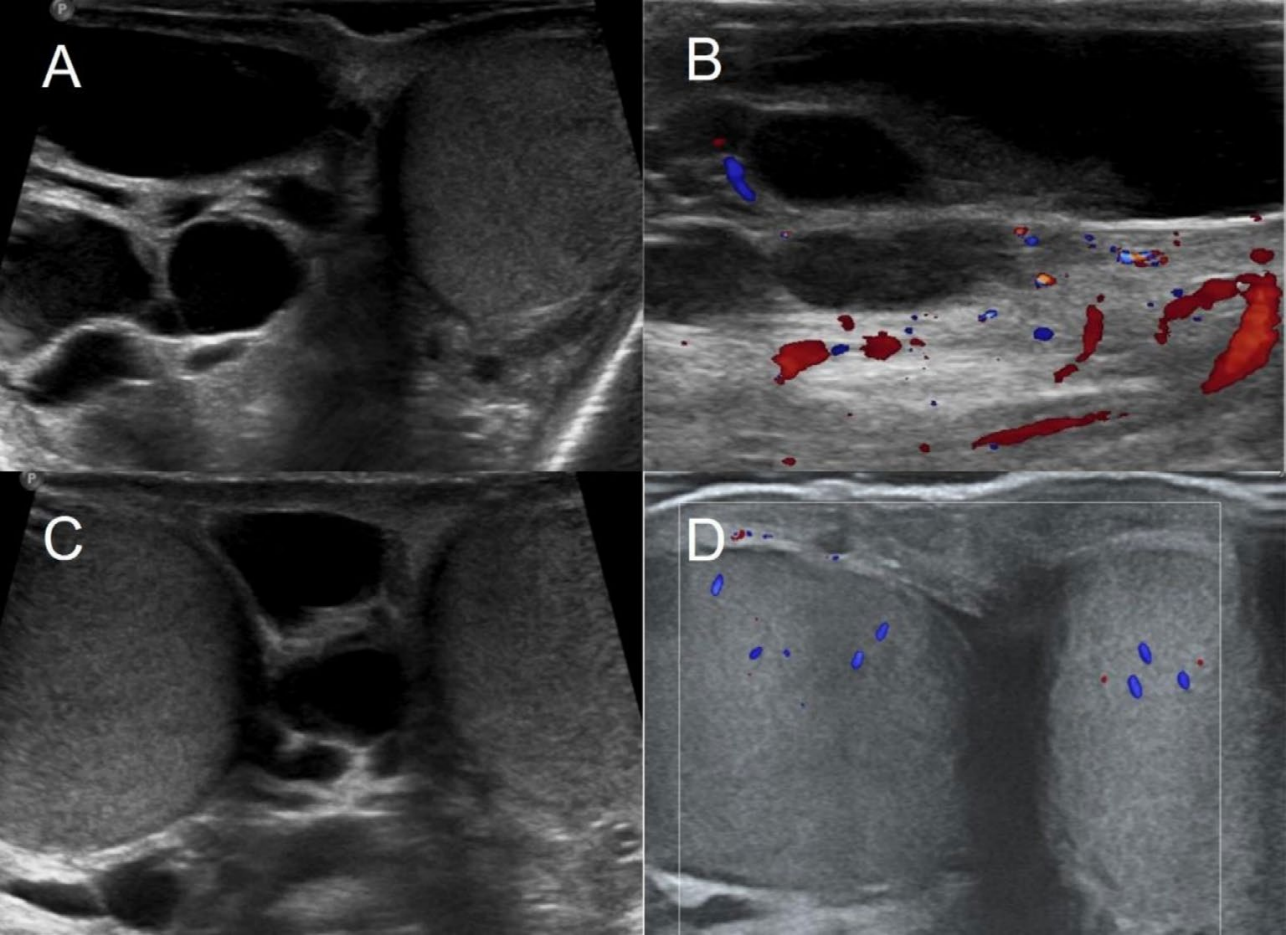
Ultrasound examination. (A–C) Are the preoperative color ultrasound images. (D) Shows the color ultrasound image obtained during the one‐year postoperative follow‐up.

**FIGURE 3:**
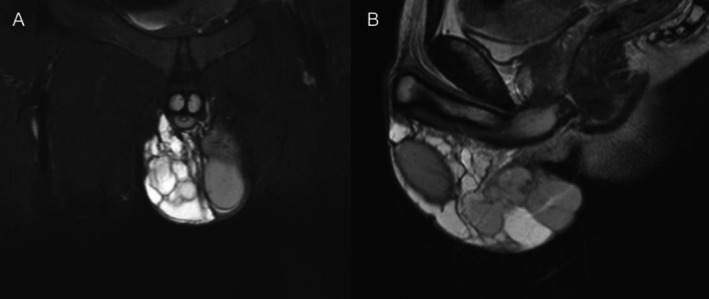
Coronal and sagittal magnetic resonance imaging. The polycystic structure measured 9.3 × 5.7 × 10.9 cm. The right testis and epididymis were compressed, but their shape and size were normal. (A) Coronal plane; (B) Sagittal plane.

The patient is 174 cm tall and weighs 54 kg. The external genitalia are developing normally. The right scrotum was irregularly lump‐like, protruding, and soft in texture with local tenderness. The right testicle and epididymis could not be palpated. The skin was undamaged, and there were no abnormalities in the left scrotum. The patient's temperature was normal. There were no abnormal findings in the laboratory tests (Figure 1). Ultrasound examination showed that the scrotal skin, bilateral testes and epididymis, and bilateral spermatic veins were normal. Multiple anechoic masses were visible around the right testis and were distributed in a honeycomb pattern. Some of the masses had poor internal sound transmission, and they had a clear boundary with the testis. The range was large, with an upper‐lower diameter exceeding 10 cm. Color Doppler flow imaging (CDFI) revealed punctate and linear blood flow signals on the septum (Figure 2A–C). Magnetic resonance imaging (MRI) examination showed an irregular patchy abnormal signal shadow in the right scrotum measuring approximately 9.3 × 5.7 × 10.9 cm in size, with multiple septa inside and uneven signals (Figure 3).

### 2.3 Treatment and Outcome

**FIGURE 4:**
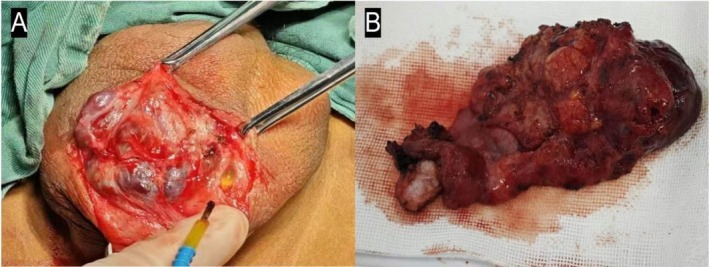
Intraoperative gross appearance during the operation. (A) Shows the cystic thickened lymphatic vessels and the cysts contained old blood clots or pale yellow lymphatic fluid. (B) Shows the complete resection of the lesion.

**FIGURE 5:**
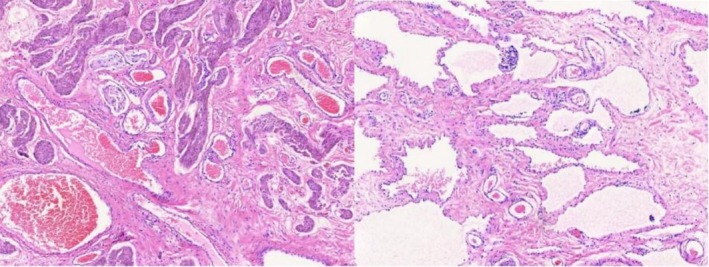
Pathologic Finding. The wall of the cyst shows endothelial cells, while the cyst cavity contains lymphocytes, red blood cells, and inflammatory cells, etc.

The patient underwent perineal mass resection, and the surgery was performed by an experienced urologist. The operation lasted for about one and a half hours. And because the entire lesion was removed intact, the amount of bleeding was only approximately 5 mL. During the operation, we observed that the scrotal shape was abnormal, and there were multiple soft, transparent, cobblestone‐like cystic masses that accumulated under the skin. These cystic masses are located between the scrotal fascia and the testicular tunica vaginalis, and they clearly demarcate from the testicular tunica. Some posterior masses were a bluish‐purple color due to inflammatory exudation after bleeding. Thickened and transparent lymphatic vessels were observed under the skin. The mass in the scrotum was a thickened and tortuous cystic lymphatic vessel, and the cystic fluid inside was clear. Old blood clots were noted in the mass (Figure 4). The masses were completely removed while the structure of the right testicle and epididymis was completely preserved. Besides, due to the large size of the masses, the skin on the right scrotum expanded. Therefore, during the operation, we removed some redundant skin to ensure appropriate skin tension and to prevent any empty spaces from remaining under the skin, to promote healing and prevent infection. According to the pathological results, the patient was diagnosed with scrotal lymphangioma. Pathological examination revealed that the cyst wall was composed of normal endothelial cells, with several cyst‐like structures present. Inside, there was homogeneous, eosinophilic lymph fluid and a small number of lymphocytes. Some areas showed red blood cell infiltration, and in some cysts and around the cyst walls, inflammatory cell infiltration was observed (Figure 5).

### 2.4 Follow‐Up

The surgical incision was fully healed 3 weeks after surgery. During the 1‐year follow‐up, there were no signs of recurrence (Figure 2D).

## 3 Discussion

Lymphangiomas are benign lesions composed of malformations of lymphatic vessels caused by the abnormal development or dilation of lymphatic vessel structures. They are also known as lymphatic malformations [1]. Lymphangiomas can be classified as congenital or acquired. In 1976, Whimster first proposed that the cause of congenital lymphangioma was due to the inability of primitive lymphatic pools in the deep subcutaneous layer to communicate with the lymphatic system during fetal development, resulting in congenital lymphatic reflux disorders [6]. Congenital lymphangiomas are commonly diagnosed in adolescents and children, with half present at birth and 90% diagnosed before the age of two. Acquired lymphangiomas are secondary to lymphatic vessel infection or local injury [7]. They are relatively rare and more common in adults [4, 5].

Based on histopathological characteristics, lymphangiomas can be divided into capillary lymphangioma, cavernous lymphangioma, and cystic lymphangioma [8]. The cysts usually contain lymphatic fluid. In our patient, cystic lymphangioma was confirmed by pathology. The primary clinical manifestation of cystic lymphangioma is painless scrotal swelling that generally progresses slowly. When a patient suddenly experiences scrotal swelling with pain, secondary factors such as intracystic hemorrhage, trauma, infection, or excessive lymphatic fluid production should be suspected [9, 10], In this case, the patient had no history of trauma. During the operation, signs of old hemorrhage were observed in some of the resected specimens. It is possible that symptoms were acutely aggravated due to exercise or personal compression.

Cystic lymphangioma should be differentiated from diseases such as testicular and spermatic cord hydroceles, varicocele, and inguinal hernia. When symptoms are acutely aggravated, differentiation from scrotal emergencies such as testicular torsion should be undertaken. Concurrently, cystic lymphangioma should be differentiated from scrotal soft tissue tumors such as lipomas and leiomyomas [10, 11]. Therefore, due to the rarity of this disease, clinicians often tend to misdiagnose it as other epididymal space‐occupying lesions.

When a patient shows clinical symptoms, an ultrasound examination is the first‐line diagnostic modality. Multichambered cysts in the scrotum can be observed on ultrasound. Color Doppler flow imaging will sometimes detect blood flow signals on the cyst wall. If there is bleeding in the cysts, the echoes of different cysts will vary. MRI is another useful technique. Cystic lymphangioma shows a low signal on T1‐weighted images and a high signal on T2‐weighted images. When there is intracystic hemorrhage, the bleeding will show a lower signal on T2‐weighted images. In addition, MRI can clearly define the scope of the lesion [9, 10].

The treatment goal of surgery for lymphangioma is to completely remove all diseased tissues. A study by Alqahtani et al. found that the recurrence rate after complete resection of the lesion was 17%–22%, while the recurrence rate after incomplete resection was as high as 35%–64% [2]. The difficulty of removing the lesion is not significant, but the key challenge and focus of surgical treatment for this disease lie in the complete and thorough removal of the lesion. If the basal lesion remains, the probability of recurrence in the future will significantly increase. As Patoulias et al. have stated, for both adults and children, if the lesion is not completely removed, the recurrence of the lesion is the most common complication [12]. Sclerosing agent injection treatment is another mainstream treatment option due to its high success rate, low recurrence rate, and low complication rate. Commonly used sclerosing agents include OK‐432 (a preparation composed of heat‐treated and freeze‐dried group A hemolytic Streptococcus and penicillin), bleomycin, peplomycin, etc. Nonselective receptor blockers such as propranolol are currently being studied for their efficacy in treating lymphangiomas. The risk of complications caused by these classes of drugs is low [13]. However, our medical center does not offer the corresponding treatment methods.

After identifying the treatment needs of the patient and clarifying the scope of the lesion through ultrasound and MRI examinations, a surgical resection treatment plan was developed. The diseased tissue was removed as completely as possible, and postoperative pathological results confirmed the diagnosis of lymphangioma. At the 1‐year follow‐up after the operation, no signs of recurrence were found.

## 4 Conclusion

Lymphangioma is a benign tumor caused by lymphatic malformation that can occur anywhere in the body, but its occurrence in the scrotum is extremely rare. This requires clinicians to have a full understanding of the disease. Scrotal ultrasound and MRI are useful for identifying and clarifying the scope of the lesion. Recurrence is reduced by completely removing the diseased tissue, and the final diagnosis should be confirmed by pathological examination.

## Author Contributions

**Sen Yan:** formal analysis, investigation, resources, writing – original draft, writing – review and editing. **Yi‐Feng Jiang:** writing – review and editing. **Dan Chen:** writing – review and editing.

## Ethics Statement

The authors have nothing to report.

## Consent

Written informed consent was obtained from the patient to publish this report in accordance with the journal's patient consent policy.

## Conflicts of Interest

The authors declare no conflicts of interest.

## Data Availability

If necessary, the relevant clinical data mentioned in this article can be obtained from the first author of the article.
